# An Integrated Approach for a New Pattern in Pediatric Primary Care: Interaction Mediation for Active and Efficient Medical Consultations

**DOI:** 10.3389/fped.2020.00530

**Published:** 2020-09-04

**Authors:** Jessica Ranieri, Federica Guerra, Eleonora Cilli, Dina Di Giacomo

**Affiliations:** Department of Life, Health and Environmental Sciences, University of L'Aquila, L'Aquila, Italy

**Keywords:** children, pediatric primary care, emotional distraction, adjuvant psychology, psychological segment

## Abstract

**Introduction:** This study analyzed the impact of an innovative integrated approach in pediatric care on children's behavior and cooperation during care.

**Methods:** The participants included 75 children aged 3–8 years (30 girls and 45 boys) recruited from a pediatric surgery department. The sample was categorized into three groups according to experimental condition: the Control, Playing, and Interaction groups.

**Results:** A one-way ANOVA revealed significant differences in cooperation between the three groups [*F*_(2,1)_ = 5.52; *p* = 0.006]. A *post-hoc* analysis showed better performance in the Interaction group (*p* = 0.002) compared to the Control group during clinical care. The Control group also showed less cooperation compared to the Playing group (*p* = 0.009).

**Conclusions:** The findings indicate that distraction before medical care can positively influence children's behavior, increasing their cooperation in medical settings. Future studies could objectively analyze physiological changes in children during medical care to better relieve anxiety and provide them with more efficient care.

## Introduction

Recent scientific research and advances in medicine have resulted in epidemiological changes marked by an increasing focus on patients' subjective perceptions of their physical, psychological, and social functioning as well as their well-being as a primary goal of clinical care. A systematic approach in pediatrics is needed to better manage children's emotional reactivity as they demonstrate high levels of anxiety, fear, and stress when they need clinical care. Furthermore, experiencing distress during medical procedures in childhood can lead to somatic symptoms later in life (1), elevated pain experiences and procedural fear, and subsequent behavioral avoidance of medical situations. This impact is observed both during consultation (i.e., longer time, low adherence to medical settings) and in lifestyle modeling related to distress procedures in infancy (2, 3).

Several studies have investigated the emotional impact of pediatric care with a focus on preoperative and postoperative surgery and have found individual differences in children's and parents' likelihood of responding negatively to surgical procedures. Poor psychological preparation for surgery can result in emotional distress and trauma for both children and their families. A recent systematic review (4) has reported various interventions that have been attempted by clinicians and researchers to reduce the prevalence, severity, and impact of preoperative anxiety, given its potential negative effects. Such interventions include the use of preoperative sedative medications, psychological preparation programs, and complementary therapies such as storybook reading, age-appropriate teaching interventions, and the use of child life specialists. Non-pharmacological treatments have proven to be somewhat effective in reducing anxiety; interventions such as clown doctors, hypnosis, low sensory stimulation, audio-visuals, and handheld video games might help to encourage children to cooperate during induction of general anesthesia, optimizing surgical care (5–8). More recently, health-related quality of life (HrQoL), defined as “a multidimensional construct covering physical, emotional, mental, social, and behavioral components of well-being and function as perceived by patients and/or other observers” (9) (p. 344), has gained attention as a health outcome in clinical studies, representing children's adaptation as a process accounting for resiliency and variability in specific indicators, leading to positive outcomes in terms of mental health (10). In two randomized controlled trials, the patient-reported HrQoL applied within medical primary care favored the patient–doctor communication and increased detection of mental health concerns and referral to mental health specialists (11, 12).

Behavioral and emotional reactions can impede the adequacy and efficacy of therapies for children. Psychological interventions are increasingly being considered to support a range of healthcare delivery needs (13) and have shown promising results in improving mood, reducing stress, and encouraging communication among children on the autism spectrum during rehabilitation.

Previous studies have already investigated anxiety and distress in children in hospital settings and during highly stressful medical and surgical procedures such as pre- and postoperative preparation, medication administration, biological exams, and endoscopic procedures. The research outcomes are productive and promising in terms of benefits to children and their parents. However, there is lack of research on the burden of anxiety in pediatric primary care settings and its influence on consultation outcomes, as well as on children's emotional experience in medical settings during infancy. Accordingly, the present study presents a framework for observing children's initial response and behavior in pediatric primary care, with the aim of providing therapeutic benefits for children through increased focus and compliance. Specifically, the study analyzed the influence of an integrated approach and intervention involving “distractor” variables on children's behavior in a pre-medical pediatric primary care setting and its related effects on the management of children during clinical care.

## Materials and Methods

### Ethics Statement

Written informed consent was obtained from each participant, and the study adhered to the Declaration of Helsinki^1^.

### Participants

The participants of this study included 75 children aged 3–8 years (30 girls and 45 boys), along with their mothers, recruited from an ambulatory pediatric primary care department in central Italy. A total of 91 eligible children were initially invited to participate, 16 of whom declined. All participants were enrolled in the pediatric primary care department during scheduled consultations. Inclusion criteria were (a) age range, 3–8 years old; (b) normal or healthy weight indicator for body mass index (BMI); and (c) no neurological/infective/psychiatric diseases. Table 1 presents the participants' demographic data.

**Table 1:** Demographic data of sample.

	**Groups**		
	**Interaction group (*N* = 30)**	**Playing group (*N* = 25)**	**Control group (*N* = 20)**
Children's mean age (SD)	6.1 (±1.4)	6.2 (±1.8)	6.1 (±1.2)
Children's mean BMI (SD)	16.8 (±2.2)	17.0 (±1.8)	16.9 (±1.9)
Parents' mean age (SD)	36.0 (±6.2)	36.8 (±6.7)	38.9 (±5.3)

*SD, standard deviation*.

### Research Protocol

**Figure 1:**
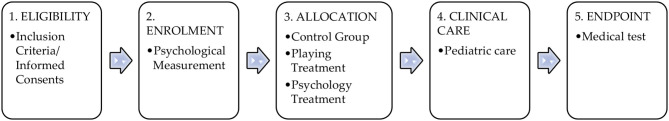
Representation of the study design and phases of the protocol.

A randomized controlled trial (RCT) was conducted, comparing treated and untreated groups, to measure the impact of the intervention on managing stress in pediatric emergency. Figure 1 shows a representation of the study design, distinguishing phases and tasks.

**Figure 2:**
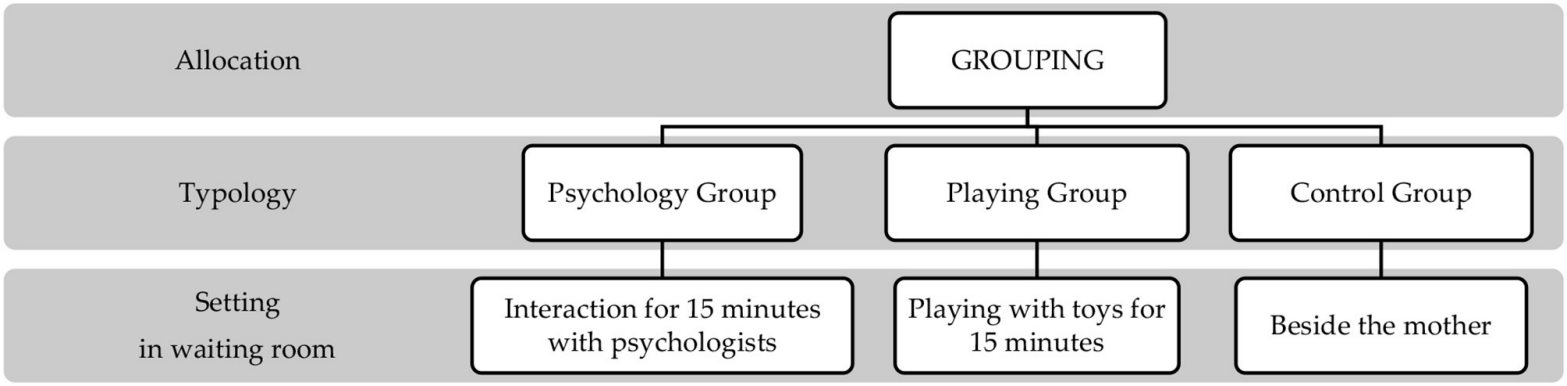
Representation of the study design and phases of the protocol.

The research design was divided into five phases: (1) eligibility, (2) enrolment, (3) allocation, (4) clinical care, and (5) endpoint. First, eligible children were approached to participate in the study. After acceptance, they were enrolled, and both children and parents were put through psychological measurements. The children were then randomly allocated into three groups: Interaction group, Playing group, or Control group (Figure 2).

After participating in the measurement phase of the research, children in the Interaction and Playing groups remained with a psychologist in a dedicated room. Children in the Interaction group were engaged by the psychologist based on personal narrative tasks for 15 min. Children in the Playing group played with toys or the psychologist for 15 min. Finally, children in the Control group participated in the measurement phase of the research but then remained with their parent in the waiting room. The individual clinical psychologist and primary care provider were blinded to the objective of the study. Data collection was conducted from April to May 2019 period. The study's methods are consistent with CONSORT 2010 guidelines (14).

### Measurement

The psychological battery was composed of standardized tests for children and parents and an experimental test for medical doctors.

#### Test of Emotion Comprehension (TEC) (15)

The TEC assesses understanding of emotions in children aged 3–11 years. Corresponding to the theoretical dimensions of emotional understanding, the test evaluates (1) recognition of emotions based on facial expressions, (2) understanding of external causes of emotions (e.g., being sad when a pet dies), (3) assigning a desire as causing an emotion, (4) understanding the role of beliefs in determining emotions, (5) understanding the influence of memory in circumstances of assessing emotional states, (6) ability to regulate emotions, (7) ability to hide or conceal an emotion, (8) understanding that a person can have mixed emotions regarding a situation (e.g., happiness and fear simultaneously), and (9) understanding the role of morality in emotions. Separate versions are provided for boys and girls, consisting of a booklet of illustrations with a story for each situation and four possible outcomes represented by emotional facial expressions. Children are asked to assign an emotion, represented by a facial expression, to the situation.

#### Children's Behavior Questionnaire (CBQ) (16)

The CBQ was developed to provide a highly differentiated caregiver report assessment of temperament in children aged 3–8 years. The instrument is based on the definition of temperament as constitutionally based individual differences in reactivity and self-regulation, influenced over time by experience. The Short Form of the CBQ was applied in this study, consisting of 36 items to evaluate (1) surgency, (2) negative affect, and (3) effortful control.

#### Behavior Thermometer (BT, Experimental Test)

The BT is a simple rapid modular tool for detecting children's cooperation during medical care. Medical doctors assign a value (range: 0–10) to a child's cooperation during his/her clinical care. The BT was implemented in a previous pilot study on 35 children in a pediatric emergency department (sample not included in the present study) and demonstrated good internal reliability (α = 0.81).

**Figure 3:**
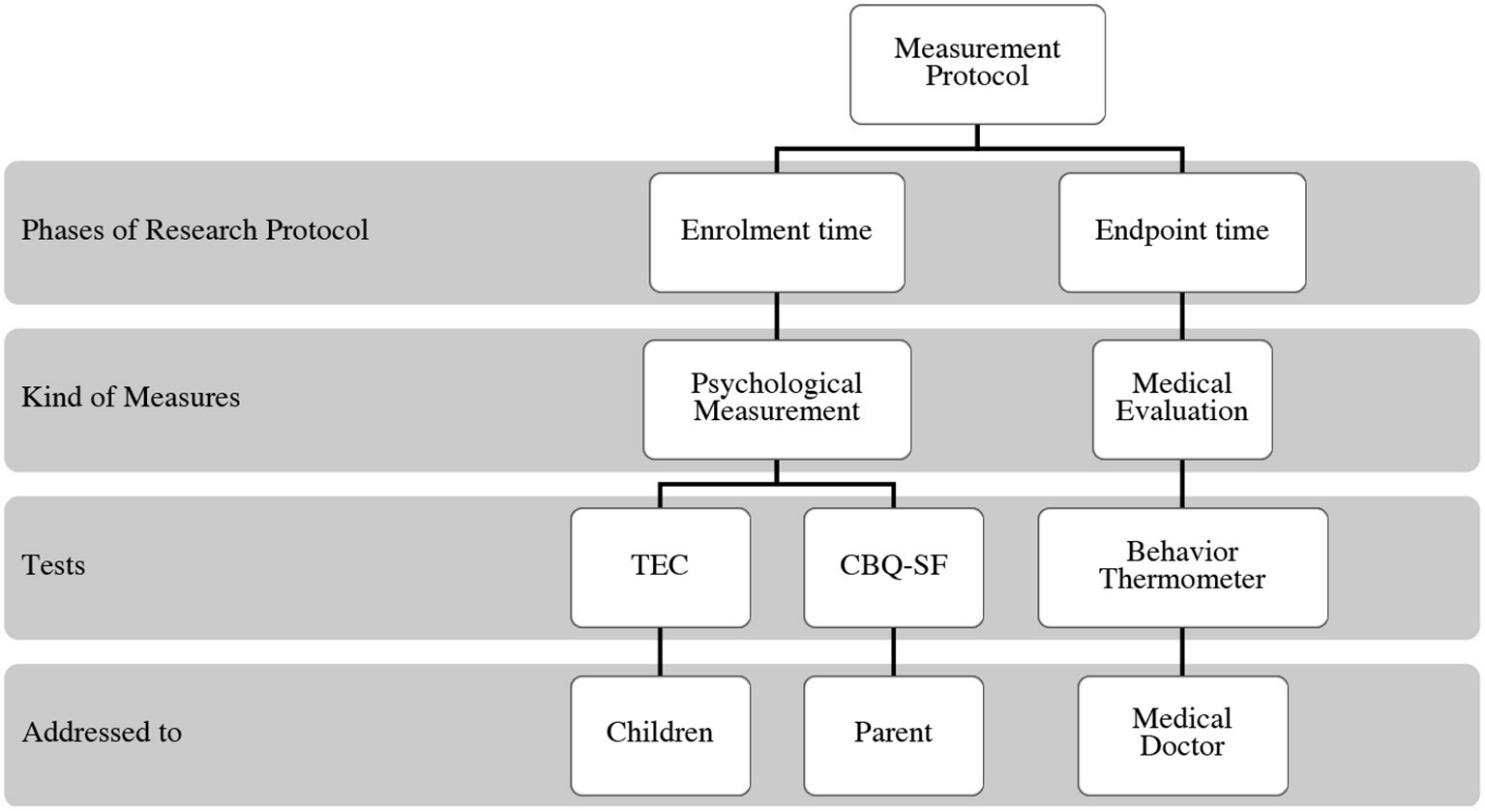
Representation of the measurement procedure.

The measurement phase of the study was divided into two, as shown in Figure 3.

### Training, Supervision, and Quality Control

The research protocol was implemented by clinical psychologists with a cognitive orientation who were trained over a 2-week period. The psychological measurements were administered by an experienced psychologist. Both psychologists were blinded to the aim of the study. Scoring of the testing protocol was conducted by external blinded judges. Medical staff members were involved in sample enrolment and managed the clinical path.

### Statistical Analyses

A randomized clinical trial (RCT) study was conducted. The study sample was stratified into three groups according to experimental condition (Control, Playing, and Interaction groups). The characteristics of the study sample were analyzed using descriptive statistics. One-way ANOVA was performed to compare categorical variables (age, psychological measures, and medical evaluations), reported as counts. All statistical analyses were performed using the SPSS software with statistical significance defined as α < 0.05.

## Results

The collected data were statistically analyzed. Table 2 presents the raw scores (means and standard deviations) obtained by the measurement tests.

**Table 2:** Raw score of measurement tests.

**Measures**	**Groups**					
	**Psychology group**		**Playing group**		**Control group**	
	**χ**	***SD***	**χ**	***SD***	**χ**	***SD***
**Children's data**						
TEC	6.0	2.1	5.5	2.2	5.8	2.0
Recognition	0.9	0.2	0.7	0.4	1.0	0.0
External cause	0.8	0.3	0.7	0.4	0.8	0.3
Desire	0.8	0.3	0.6	0.4	0.7	0.4
Beliefs	0.6	0.4	0.7	0.4	0.7	0.4
Infl. of memory	0.7	0.4	0.7	0.4	0.6	0.5
Regulation of emotion	0.4	0.5	0.4	0.5	0.2	0.4
Hiding emotion	0.5	0.5	0.5	0.5	0.7	0.4
Mixed emotions	0.3	0.4	0.2	0.4	0.2	0.4
Role of morality	0.6	0.4	0.5	0.5	0.5	0.5
**Parents' data**						
CQB	168.7	21.0	165.5	18.1	164.1	18.0
Surgency	51.6	7.9	52.3	6.7	54.0	8.0
Negative affect	51.1	11.2	49.6	7.3	53.0	8.5
Effortful control	66.8	9.8	64.5	8.3	61.1	9.1
**Medical data**						
BT	8.9	1.5	8.9	1.5	8.0	1.3

*χ, mean value; SD, standard deviation*.

One-way ANOVA to compare each group's performance on the TEC showed no significant differences in emotion comprehension. Similarly, comparison of the children's temperamental reactions in the clinical care condition showed no significant differences in reactivity or self-regulation. This preliminary data analysis showed that the sample distribution into the three research groups was composed of children with no emotional significant differences and even disorders, no sign for temperamental weakness toward adaptation behavioral ability, and no fragility to deal with new experiences or contexts.

Then, we wanted to analyze the children's behavior during consultations after exposition to three experimental conditions. One-way ANOVA comparing BT values between the three children groups revealed significant differences [*F*_(2,1)_ = 5.52; *p* = 0.006]. *Post-hoc* analysis showed better performance in the Interaction group (*p* = 0.002) than in the Control group during clinical care. The Control group also demonstrated less cooperation than the Interaction group (*p* = 0.009). No significant differences were between the Interaction and Playing groups.

Our results showed the positive impact of interaction mediation in medical consultations: the interaction with a psychologist or playing conditions can lead to the emotional wellness of children, making them pay their attention to involvement in the relationship or playing, and in turn favoring better and efficient behavioral adaptation among children to the pediatric setting. The children's higher adherence to the medical setting might prompt efficiency in primary care.

## Discussion

The aim of the study was to investigate the potential role of interaction mediation in medical consultations for a new pattern of pediatric primary care as part of an integrated approach in innovative medicine.

We established interesting findings that highlighted the role of interaction mediation within the patient-centered approach, overcoming the challenges of disease-centered care; the mediating effect of interaction as a distractor toward pediatric consultation for children facilitated an improvement in the child–doctor communication. The medical doctor reported a higher adherence among children to the medical setting, and more efficiency with regard to the consultation time. Our findings demonstrated that pediatric primary care could be improved by modeling the pre-medical setting and integrating it into the consultation: Psychological distress and/or fear for medical interaction among children might be decreased through psychological mediation and/or play as a mediator; our findings showed a positive influence on children's behavior, making them more cooperative in a medical setting, leading to more adequate and efficient consultations. The proposed approach of integrated care, applying adjuvant psychology for innovative and personalized medicine, could improve the efficacy of pediatric primary care. Due to age, children tend to be uncooperative patients, even more so if they are focused on their fear. The mediation of interaction intervention in which the children focus on their relationship with an adult, making their fear of medical evaluation secondary, facilitates the adaptation process in children, making the pediatric care setting less frightening. Our findings are relevant in that they demonstrated that distractive strategies improved doctor–child cooperation in clinical care. Importantly, the medical endpoint highlighted the low management of children in the Control group who remained with their parent in the waiting room prior to care; in contrast, the children who interacted with an adult (psychologist) or toys entered an adaptive setting in which they overlooked their own fear and anxiety. Verbal prompts (personal narrative tasks) are a powerful tool for helping children overcome their concentration on feelings and shift their attention to cognitive elaboration about themselves. Children's emotional development and parental distress were similar in all the three groups and had no effect on clinical care; on the contrary, exposure to distractor strategies elicited better and more efficient patient cooperation. Innovative medicine involves functional and efficient clinical solutions that require patients to actively participate in consultations; in this role, children can demonstrate some adaptive behavior issues, refusing to interact with medical doctors; nevertheless, it takes time to establish a patient–doctor relationship. This study showed that initial interaction with a psychologist before clinical consultation can positively influence children's adaptation to interaction in a clinical setting.

This finding is consistent with existing evidence regarding the efficacy of an integrated approach in pediatric primary care with psychologist as a mediator of active and efficient medical consultations (6–9); this approach could be useful not only in emergency and hospital settings (pre- and post-operative surgery) as already investigated (4, 5) but also in primary care. Moreover, in addition to the short-term benefit of more efficient medical care and better management of children's interaction in pediatrics, this approach could also have a long-term benefit of reducing medical anxiety among both youths and adults, aligning primary care more with patients' needs rather than positioning it as a source of anxiety regarding health and outcomes.

There are some limitations in the study's design and procedures. First, the sample size was small; a total of 75 children participated, and they were distributed into three smaller groups. Additionally, the age range of the sample was quite large (3–8 years), including children in different cognitive and emotional development stages. Future studies with larger samples and stratification by age group could increase the consistency of the findings.

Despite these limitations, this study highlighted the relevance of psychology in pediatric care settings to predict the individual risk of developing medical mistrust and fear, to facilitate clinical care, and reinforce the concept of psychological intervention as a mediator for more efficient primary health care, with active participation of children and parents. This study dealt with an emerging framework dedicated to the advanced modeling of pediatric primary care in order to set an integrated approach applying adjuvant psychology in investigating children's behavior during scheduled consultations. Considering the study's limitations, a more extensive study will be conducted in the future to deeply address clinical outcomes, psychological, social, and cultural variables that can facilitate or hinder primary care services, and factors that facilitate better pediatric consultations for health promotion and preventive health policy. Future studies should address the topic of designing a clinical trial to measure and quantify the effective impact in medical consultations in terms of quality of detection data as well as longitudinal researches for the evaluation of influence of adjuvant psychology in pediatric primary care, leading to better management of children/patient–doctor communication.

## Data Availability Statement

The raw data supporting the conclusions of this article will be made available by the authors, without undue reservation.

## Ethics Statement

The studies involving human participants were reviewed and approved by Internal Review Board of University of L'Aquila (code 16372/2019). Written informed consent to participate in this study was provided by the participants' legal guardian/next of kin.

## Author Contributions

DD: conceptualization. JR: methodology and formal analysis. FG and EC: data curation. All authors: writing, review, editing, and read and agreed to the published version of the manuscript.

## Conflict of Interest

The authors declare that the research was conducted in the absence of any commercial or financial relationships that could be construed as a potential conflict of interest.

## Footnotes

^1^https://www.wma.net/what-we-do/medical-ethics/declaration-of-helsinki/ (accessed March 3, 2020).
